# Patterns and Outcomes of Recurrence in Pediatric Langerhans Cell Histiocytosis

**DOI:** 10.14740/jh2197

**Published:** 2026-06-20

**Authors:** Sudipto Bhattacharya, Anuj Singh, Nita Radhakrishnan, Aditi Tulsiyan, Archit Pandharipande

**Affiliations:** a Department of Pediatric Hematology Oncology, Post Graduate Institute of Child Health, Noida, Delhi NCR, India; b Current address: Department of Hematology, All India Institute of Medical Sciences (AIIMS), Nagpur, India

**Keywords:** Langerhans cell histiocytosis, First recurrence, Clinical profile

## Abstract

**Background:**

Langerhans cell histiocytosis (LCH) is a rare neoplasm with a heterogeneous clinical spectrum ranging from isolated lesions to multisystem disease, with recurrence reported in up to 50–60% of patients. Data on outcomes of recurrent LCH from resource-limited settings remain limited.

**Methods:**

We report a 12-year retrospective review of children (1–18 years) with biopsy-proven relapsed LCH treated between January 2014 and December 2025. Clinical characteristics, recurrence patterns, treatment, and long-term outcomes were analyzed. All patients had received first-line therapy according to LCH-III protocol. Salvage treatments included cladribine-based chemotherapy, lenalidomide–dexamethasone (Len-Dex), or surgery for isolated lesions. Follow-up was updated to December 31, 2025.

**Results:**

Seven patients with a median age of 22 months at diagnosis (range 6–57) were included. Four had risk-organ involvement at presentation. Diabetes insipidus was present in 4/7 (57%). The median time to first recurrence was 25.8 months (range 6–104). Recurrence involved bone alone in four patients and bone with skin in three patients; none had risk-organ involvement at relapse. Three patients experienced multiple recurrences. Salvage therapy included cladribine-based chemotherapy (n = 6), Len-Dex (n = 2), and surgery (n = 1). At a median follow-up of 114 months (range 63–169), overall and event-free survival were 100%. Long-term sequelae included persistent diabetes insipidus (3/4), sclerosing cholangitis (n = 1), and sensorineural hearing loss (n = 1).

**Conclusions:**

Children with recurrent LCH in this cohort demonstrated good long-term outcomes irrespective of the salvage treatment strategy. Len-Dex represents a feasible, cost-effective therapeutic option in resource-limited settings where access to targeted therapy is limited.

## Introduction

Langerhans cell histiocytosis (LCH) is a rare inflammatory myeloid neoplasm characterized by clonal proliferation of pathological dendritic cells expressing CD1a and CD207. The disease demonstrates a highly heterogeneous clinical spectrum, ranging from isolated self-limiting bone lesions to aggressive multisystem involvement affecting organs such as the liver, spleen, lungs, lymph nodes, skin, and the central nervous system, particularly the hypothalamic–pituitary axis [1].

Disease classification is primarily based on the extent of involvement and the presence of risk-organ disease. Patients with multisystem disease involving the liver, spleen, or hematopoietic system are known to have significantly poorer outcomes compared with those with single-system disease or multisystem disease without risk-organ involvement. Over the past two decades, collaborative studies conducted by the Histiocyte Society have substantially improved treatment outcomes. The LCH-III trial established vinblastine combined with prednisolone as the standard frontline therapy and demonstrated that prolongation of treatment to 12 months significantly reduces disease reactivation in patients with multisystem disease [2].

Despite these therapeutic advances, disease recurrence remains a major challenge in the management of LCH. Reactivation rates ranging from 27% to 60% have been reported, depending on disease extent and response to therapy. Recurrences may occur months or years after completion of therapy and may involve previously affected or new organ systems. Consequently, the management of recurrent or refractory LCH remains heterogeneous, with no universally accepted salvage regimen [3, 4].

Combination chemotherapy with cladribine and cytarabine has shown efficacy in refractory multisystem disease but is associated with considerable toxicity [3]. Other therapeutic approaches including vincristine-based regimens, clofarabine monotherapy, hematopoietic stem cell transplantation, and targeted therapies directed at mitogen-activated protein kinase (MAPK) pathway mutations have been explored. The discovery of activating mutations in the MAPK pathway, particularly BRAF-V600E, has further transformed the therapeutic landscape by enabling the use of targeted agents such as BRAF and MEK inhibitors [5].

In low- and middle-income countries, however, access to molecular diagnostics, targeted therapies, and transplantation remains limited. Under such circumstances, alternative and cost-effective treatment strategies are often required. Immunomodulatory therapy with lenalidomide combined with dexamethasone has recently emerged as a potential option for relapsed or refractory LCH, with encouraging responses reported in small clinical series [4].

Data describing the clinical characteristics and outcomes of recurrent LCH from resource-limited settings remain scarce. Understanding patterns of recurrence and treatment responses in such environments is therefore important for guiding therapeutic decision-making. In this study, we describe the clinical profile, treatment approaches, and long-term outcomes of pediatric patients with recurrent LCH treated at a tertiary pediatric hematology–oncology center in North India.

## Materials and Methods

This retrospective observational study was conducted at the Department of Pediatric Hematology Oncology, Post Graduate Institute of Child Health, Noida, India, a tertiary referral center for pediatric hematologic and oncologic disorders. The medical records of children diagnosed with LCH who subsequently developed disease recurrence were reviewed.

Children aged 1–18 years with biopsy-proven LCH and disease recurrence from January 2014 to December 2025 were included. The diagnosis of LCH was established by histopathological examination of affected tissue with immunohistochemical confirmation of CD1a positivity. Patients were included if they had documented recurrence following completion of initial therapy and if adequate clinical data were available for analysis. Patients with a single episode of LCH without recurrence or those with incomplete medical records were excluded. Institutional ethics committee approval for reporting outcome based on chart review was obtained prior to data collection (Study Protocol Number: 2023-10-EM-43, Institute: Post Graduate Institute of Child Health, Noida).

Clinical and demographic data were extracted from hospital records and electronic medical databases using a structured data collection form. Information collected included age at initial diagnosis, sex, risk stratification at presentation according to the LCH-III classification, presence of diabetes insipidus at diagnosis, treatment received at initial presentation, response to therapy, time to recurrence from initial diagnosis, clinical manifestations at recurrence, sites of disease involvement, treatment administered at recurrence, number of recurrence episodes, and long-term clinical status including late effects.

Disease recurrence was defined as the reappearance of clinical or radiologic evidence of active LCH following complete remission or a period of disease control lasting at least 3 months after completion of therapy. All patients received first-line therapy with vinblastine and prednisolone according to the LCH-III protocol. Induction therapy was followed by continuation therapy as per risk stratification.

Treatment strategies employed at recurrence included salvage chemotherapy with cladribine (2-chlorodeoxyadenosine) and cytarabine, modified low-dose cladribine–cytarabine regimens, lenalidomide combined with dexamethasone, or localized surgical excision for selected patients with isolated bone lesions [3].

In the standard salvage protocol, cladribine was administered at a dose of 9 mg/m^2^/day for 5 days along with cytarabine at 1 g/m^2^/day for 5 days. In the modified salvage regimen, cladribine was administered at 5 mg/m^2^/day for 5 days with cytarabine at 100 mg/m^2^/day for 4 days. The lenalidomide–dexamethasone regimen consisted of six cycles of oral lenalidomide administered for 21 days of a 28-day cycle (2.5 mg for children weighing less than 15 kg and 5 mg for those weighing more than 15 kg) in combination with dexamethasone administered at a dose of 0.8 mg/kg on days 1, 8, 15, and 21 [4]. Response assessment was based on clinical evaluation in conjunction with 18F-fluorodeoxyglucose positron emission tomography imaging. Sites demonstrating persistent or suspicious metabolic activity were further evaluated with histopathological confirmation through biopsy when required.

Patients were followed in the institutional survivorship clinic following completion of therapy. Outcome measures included overall survival, event-free survival following recurrence, number of recurrence episodes, and treatment-related late effects. Clinical follow-up for the present analysis was updated until December 31, 2025.

## Results

### Patient characteristics at diagnosis

Seven consecutive patients fulfilled the inclusion criteria for analysis. The median age at initial diagnosis was 22 months (range 6–57 months). Male-to-female ratio was 6:1. Risk stratification at diagnosis according to the LCH-III classification revealed multisystem disease with risk-organ involvement in four patients, multisystem disease without risk-organ involvement in two patients, and single-system multifocal bone disease in one patient. The median age of patients with risk-organ–positive disease was 16 months. The detailed clinical characteristics, treatment strategies, and outcomes of the seven patients are summarized in Table 1.

**Table 1:** Clinical Characteristics and Outcomes of Pediatric Patients With Recurrent Langerhans Cell Histiocytosis

Patient	Sex	Age at initial diagnosis (months)	Time to first recurrence (months)	Initial risk stratification	Diabetes insipidus at diagnosis	Disease manifestation at recurrence	Treatment at recurrence	Complications during treatment	Number of recurrences	Current status	Follow-up from diagnosis (months)
1	Male	23	6	Multisystem RO+	Yes	Multifocal bone and skin involvement; MRI brain - pituitary stalk thickening	Cladribine + cytarabine (LCH-S protocol)	Febrile neutropenia - two episodes Respiratory support needed	1	CR2	114
2	Male	13	28	Single-system multifocal bone	No	Multifocal bone lesions	Cladribine + cytarabine in initial recurrence; Lenalidomide + dexamethasone in later recurrences	Initial recurrence: 1) Febrile neutropenia - three episodes; 2) Transfusion support Later recurrences: nil	3	CR4	169
3	Male	10	14	Multisystem RO− sclerosing cholangitis	No	Multifocal bone and skin involvement	Cladribine + cytarabine	Febrile neutropenia - two episodes	1	CR2	102
4	Male	16	18	Multisystem RO−	No	Multifocal bone lesions	Cladribine + cytarabine (LCH-S protocol)	Febrile neutropenia - one episode	1	CR2	135
5	Female	57	39	Multisystem RO−	Yes	Single-site bone lesion	Cladribine + cytarabine (LCH-S protocol)	Febrile neutropenia - one episode	1	CR2	112
6	Male	43	22	Multisystem RO−	Yes	Multifocal bone and skin involvement; MRI brain - absence of pituitary bright spot	Lenalidomide + dexamethasone	Nil	2	CR3	63
7	Male	8	42	Multisystem RO+	Yes	Single-site bone lesion	Surgery; cladribine + cytarabine	Nil	3	CR3	132

Diabetes insipidus was present at the time of diagnosis in four patients (57%). All patients received systemic therapy with vinblastine and prednisolone according to the LCH-III protocol. Response assessment following induction therapy demonstrated either absence of active disease or significant clinical improvement in all patients. Each patient subsequently completed 12 months of continuation therapy.

### Characteristics of disease recurrence

The median time to first recurrence from the initial diagnosis was 25.8 months (range 6–104 months). At recurrence, disease involvement most commonly affected the skeletal system. Two patients presented with single-site bone lesions, two had multifocal bone disease, and three demonstrated combined bone and skin involvement. Patients were systematically evaluated for central nervous system (CNS) involvement, including assessment for endocrinopathies and clinical features of neurodegenerative LCH. None of the patients had risk-organ involvement at the time of recurrence. Notably, none of the patients developed new-onset diabetes insipidus preceding recurrence.

### Treatment approaches at recurrence

Treatment approaches at recurrence varied depending on disease distribution and clinical considerations. Four patients received salvage chemotherapy with cladribine and cytarabine. Two patients were treated with a lenalidomide–dexamethasone regimen, while one patient with an isolated bone lesion underwent surgical excision without systemic therapy. During follow-up, three patients experienced more than one recurrence of disease. One patient developed three recurrence episodes and ultimately achieved sustained remission after treatment with cladribine–cytarabine followed by lenalidomide–dexamethasone and is currently in fourth complete remission (CR4). Two additional patients experienced two recurrences each and achieved remission following subsequent salvage therapy, including lenalidomide-based treatment in one case and cladribine–cytarabine in another. Despite multiple relapse episodes, all three patients remain alive and in continued remission at the last follow-up. Two patients (P2 and P6) continue to lenalidomide–dexamethasone following achievement of remission and remain under regular clinical follow-up. BRAF V600E mutation testing was performed in these two patients at recurrence, and both were found to be negative for the mutation.

Notably, among the six recurrence episodes managed with intensive salvage therapy, all required hospital admission for treatment and management of complications. Five patients developed febrile neutropenia (median 1.8 episodes) within the initial 3 months of therapy, and one patient required respiratory support. In contrast, both patients treated with lenalidomide–dexamethasone tolerated therapy well in the outpatient setting, with no significant treatment-related toxicity.

Surgical management was not feasible in cases with multifocal skeletal involvement. Among the two patients with single-site bony recurrence, both lesions were located at CNS-risk sites (frontal and zygomatic bones), where local therapy alone was considered inadequate; therefore, systemic treatment was administered.

### Survival and long-term outcomes

At a median follow-up of approximately 114 months from initial diagnosis (range 63–169 months) as of December 31, 2025, all seven patients were alive and in complete remission. The event-free survival following recurrence was 100%, with a median follow-up of approximately 6 years from the time of first recurrence.

Long-term complications were limited. Persistent diabetes insipidus was noted in three of the four patients who had the condition at diagnosis and continued to require ongoing desmopressin therapy during follow-up. In addition, one patient had sclerosing cholangitis from diagnosis, which is being managed conservatively with ongoing close gastroenterological evaluation. He has stable liver function and currently does not require liver transplantation. One patient developed bilateral sensorineural hearing loss, for which the child is currently managed with hearing aids and regular audiological follow-up. All children were attending school and were able to participate in age-appropriate daily activities. No additional late endocrine complications were observed, including growth failure, pubertal delay, or other hypothalamic–pituitary dysfunction. Furthermore, there were no cases of secondary malignancy or severe long-term treatment-related toxicity during the follow-up period. These findings suggest that, in addition to excellent disease control, long-term functional and developmental outcomes were favorable in this cohort of patients with recurrent LCH.

## Discussion

LCH is now recognized as an inflammatory myeloid neoplasm characterized by activation of the MAPK pathway. Activating mutations in genes such as BRAF and MAP2K1 are present in a large proportion of patients and are believed to drive pathological proliferation of dendritic cells and inflammatory cytokine signaling. These discoveries have significantly changed the conceptual understanding of LCH and have led to the development of molecularly targeted therapies [6–8].

Over the past two decades, cooperative clinical trials conducted by the Histiocyte Society have established standardized treatment protocols for pediatric LCH. The LCH-III trial demonstrated that vinblastine combined with prednisone constitutes an effective first-line regimen and that extending therapy from 6 to 12 months significantly reduces disease reactivation, particularly in patients with multisystem disease. Subsequent trials, including the LCH-IV study, further evaluated risk-adapted therapeutic approaches and intensified treatment for high-risk disease. Despite these advances, disease recurrence remains common, especially among patients with multisystem involvement at presentation.

In our cohort, the majority of patients who experienced recurrence had multisystem disease at diagnosis, consistent with previous observations that initial disease burden is an important predictor of relapse. Interestingly, none of the patients in our series demonstrated risk-organ involvement at recurrence, suggesting that disease behavior at relapse may differ from that observed at initial presentation. Patients with multiple relapses demonstrated a similar pattern, with recurrences predominantly involving the skeletal system. We also highlight that durable disease control can be achieved even in patients with repeated recurrences using sequential salvage therapies.

Historically, treatment options for recurrent or refractory LCH have included cytotoxic chemotherapy regimens such as cladribine and cytarabine, which have shown substantial activity in high-risk multisystem disease but are associated with considerable hematologic toxicity. In our cohort, modified cladribine-based salvage therapy achieved effective disease control without major treatment-related toxicity [2].

An important observation from our study is the favorable outcome observed with lenalidomide and dexamethasone therapy in patients with multiple recurrences. Uppuluri and colleagues first described the use of this regimen in relapsed or refractory LCH and reported excellent remission rates with limited toxicity, suggesting that immunomodulatory therapy may represent a feasible alternative to intensive chemotherapy in selected patients. Lenalidomide exerts immunomodulatory, anti-angiogenic, and anti-inflammatory effects, which may be beneficial in a disease characterized by both neoplastic and inflammatory components. In addition, the regimen can often be administered in an outpatient setting, making it particularly attractive in resource-limited environments.

Recent advances in molecular biology have led to the development of targeted therapies directed against mutations in the MAPK signaling pathway. Approximately 50–60% of patients with LCH harbor BRAF-V600E mutations, which are associated with more aggressive disease and higher rates of treatment resistance. Targeted therapies, including BRAF inhibitors (vemurafenib, dabrafenib) and MEK inhibitors such as trametinib, have demonstrated encouraging responses in patients with relapsed or refractory LCH, with several studies reporting rapid clinical improvement and high response rates [9–12].

However, the high cost of targeted agents and limited availability of molecular diagnostics restrict their widespread use in many low- and middle-income countries [13–15]. In such settings, alternative treatment strategies that are effective, less toxic, and affordable are needed. Immunomodulatory therapy with lenalidomide and dexamethasone has shown promising activity in relapsed or refractory LCH and may represent a feasible and cost-effective option in resource-limited settings [4].

The good survival observed in our study likely reflects several factors, including early recognition of recurrence, prompt initiation of therapy, and careful long-term follow-up in a specialized pediatric oncology center. Importantly, our findings demonstrate that durable remission can still be achieved with conventional and immunomodulatory treatment strategies even in the absence of targeted therapy.

However, the rapidly evolving landscape of LCH treatment suggests that integration of molecularly targeted therapies into existing treatment algorithms may further improve outcomes, particularly for patients with refractory multisystem disease or those harboring actionable MAPK pathway mutations. Prospective studies evaluating combinations of targeted agents with conventional chemotherapy will be essential to define optimal therapeutic strategies.

### Limitations

This study has several limitations that should be acknowledged. First, the retrospective design based on chart review may introduce inherent biases related to data completeness and documentation. Although every effort was made to ensure comprehensive data retrieval and we are confident that no eligible patient with recurrent LCH during the study period was missed, the retrospective nature of the analysis limits the ability to control for potential confounding factors.

Second, the sample size is small, reflecting both the rarity of LCH and the single-center design of the study. Consequently, the findings should be interpreted with caution and may not be generalizable to larger populations.

Importantly, the majority of patients in our cohort experienced low-risk relapses without risk-organ involvement. Therefore, the favorable outcomes observed in this series may not necessarily be extrapolated to patients with high-risk or risk-organ–positive relapses, who are known to have a more aggressive disease course and may require more intensive therapeutic approaches. The treatment strategies used in our cohort, including cladribine-based salvage therapy and lenalidomide–dexamethasone, may not demonstrate similar efficacy in patients with high-risk recurrent disease.

Prospective multicenter studies including larger cohorts and patients with diverse risk profiles are needed to better define optimal treatment strategies for recurrent LCH.

### Conclusion

In this single-center cohort of pediatric patients with recurrent LCH, long-term outcomes were excellent with currently available treatment approaches. Salvage chemotherapy with cladribine–cytarabine combinations and lenalidomide-based therapy both achieved sustained remission. These findings suggest that less intensive therapeutic strategies may represent viable options in resource-limited settings. Early detection of recurrence and prompt initiation of therapy remain critical for achieving favorable outcomes.

## Data Availability

The data supporting the findings of this study are available from the corresponding author upon reasonable request.
